# Do Deepfakes Adequately Display Emotions? A Study on Deepfake Facial Emotion Expression

**DOI:** 10.1155/2022/1332122

**Published:** 2022-10-18

**Authors:** Juan-Miguel López-Gil, Rosa Gil, Roberto García

**Affiliations:** ^1^LSI Department, University of the Basque Country, Donostia-San Sebastián, Spain; ^2^Department of Computer Science and Engineering, Universitat de Lleida, Lleida, Spain

## Abstract

Recent technological advancements in Artificial Intelligence make it easy to create deepfakes and hyper-realistic videos, in which images and video clips are processed to create fake videos that appear authentic. Many of them are based on swapping faces without the consent of the person whose appearance and voice are used. As emotions are inherent in human communication, studying how deepfakes transfer emotional expressions from original to fakes is relevant. In this work, we conduct an in-depth study on facial emotional expression in deepfakes using a well-known face swap-based deepfake database. Firstly, we extracted the photograms from their videos. Then, we analyzed the emotional expression in the original and faked versions of video recordings for all performers in the database. Results show that emotional expressions are not adequately transferred between original recordings and the deepfakes created from them. High variability in emotions and performers detected between original and fake recordings indicates that performer emotion expressiveness should be considered for better deepfake generation or detection.

## 1. Introduction

Recent technological improvements have made it simple to construct “deepfakes” and hyper-realistic videos that use face swaps and leave little evidence of alteration [1]. Artificial intelligence (AI) apps blend, replace, and superimpose photos and video clips to generate phony videos that appear legitimate [2]. Without the approval of the individual whose image and voice are involved, deepfake technology can manufacture any kind of material [3]. The extent, scale, and sophistication of the technology involved in deepfakes are game-changing, as essentially anyone with a computer can create fake movies that are virtually indistinguishable from legitimate media [4]. Most early examples of deepfakes focused on joke videos about well-known individuals. However, risks are emerging as they are being used for revenge porn, bullying, fake video evidence in courts, political sabotage, terrorist propaganda, blackmail, market manipulation, and fake news [2].

The human face can express emotions faster than people can explain or even comprehend their sentiments, making facial expression one of the most direct ways humans transmit their emotions [5]. Automatic facial expression recognition (FER) is becoming a hot topic in academia.

Although most of these systems aim to recognize only a small number of prototypical emotional expressions, much progress has been made in developing computer systems that analyze this sort of human communication [6–8]. Many recent efforts on facial emotion recognition based on facial expressions have used deep learning to solve the problem, whether in static photos or dynamic video recordings [9–13]. Emotions in FER are becoming growingly important in many fields, such as health [14].

Literature on deepfakes and emotions is scarce. To the authors' knowledge, the only works that specifically tackle audio-visual deepfake detection using affective cues are [15, 16], and [17]. Moreover, we could not find specific studies on how well deepfakes express emotions.

How well emotions are displayed in deepfakes when faces are swapped between different performers is an interesting research question. To that end, an in-depth study on facial emotional expression in deepfakes is carried out in this work. With that goal, a well-known face swap-based deepfake database is used. The photograms from their videos were extracted first, and the emotional expression was analyzed in the original and faked versions of the video recordings for all performers in the database. Results show that emotional expressions are not adequately transferred between original recordings and the deepfakes created from them. The high variability in emotions and performers detected between original and fake recordings indicates that performer emotion expressiveness should be considered for better deepfake creation.

The structure of this document is as follows: the related work section presents studies found in the literature on deepfakes, FER, and emotion recognition. The methods section describes used databases and introduces the method used to categorize emotional photograms from video recordings. The Results section shows obtained results in detail. Results are interpreted, and the discovered knowledge is displayed in the Discussion section, while the Conclusion section presents the conclusions.

### 1.1. Related Work

Although there have been numerous advanced algorithms for making realistic synthetic face films in recent years [18], most of them have not yet become widely available as open-source software tools that anybody can use. On the other hand, a much simpler method based on the work of neural image style transfer [19] has become the tool of choice beneath many deepfake videos or existing deepfake datasets. Moreover, there are several open-source implementations, such as Face2face [20] and DeepFaceLab [21]. [22] provides a comprehensive review of deepfake technologies.

The encoder and decoder are usually two convolutional neural networks that make the autoencoder. The encoder turns the face of the input target into a code vector. There is only one encoder, regardless of the individuals' identities, to ensure that identity-independent features are captured, such as facial expressions. Each identity, on the other hand, has its decoder, which uses the code to generate the face of the matching subject. In an unsupervised way, the encoder and decoder are trained in tandem using noncorresponding face sets of various participants.

Deepfakes are difficult to be identified effectively with current detection methods. Shad et al. implemented several methods to detect deepfake images and made a comparative analysis [23]. In detection models, to identify video tampering, stacked autoencoders, CNNs [24], long-short term memory (LSTM) networks [25], Siamese networks [26], or GANS have been investigated. The algorithm in [27] recognized false videos by detecting key video frames, which reduced calculation time and increased accuracy when the video featured more than one keyframes. EfficientNet-V2 has also been used with good results to detect deepfakes in large-scale fake face video datasets [28].

Multiple attempts have been made to release benchmark datasets because of the rise in AI-generated deepfake material. The amount and quality of early deepfake detection datasets, such as the UADFV dataset [29] and the DeepfakeTIMIT dataset [30], are limited. Many of these issues are addressed by the more recent FaceForensics++ [31] and the DeepFake Detection Challenge dataset [32], as well as other viable deepfake detection databases. The Google DeepFake Detection dataset [33] contains 3,068 deepfake movies created from 363 original footage of 28 agreed individuals in 16 situations. The source video recordings of 100 hired actors are included in DeeperForensics [34], while 1,000 target videos are taken from FF++. Each source identity is swapped onto 10 target videos to create 1,000 phony videos. 590 real videos and 5,639 false videos make up Celeb-DF [35]. The original videos were obtained from YouTube, and the content consisted of 59 celebrity interviews. An enhanced face swap approach is used to create the faked videos.

In terms of facial expression emotion recognition, Ekman and Friesen created a facial action coding system (FACS) to represent facial expressions using action units (AUs) [36]. They identified 30 FACS AUs, out of the 44 FACS AUs they described, that were anatomically associated with the contractions of facial muscles. Of these 30 FACS AUs, 12 correspond to the upper face and 18 to the lower face. AU scans can be done individually or in groups [6]. Human encoders can use FACS to encode all facial expressions using these 30 AUs manually. The emotional labels may be described by the AU combinations defined in the FACS. FACS has become a face behavior measurement criterion in various disciplines, including computer-based vision [37], because of its descriptive ability. Based on FACS, the emotion facial action coding system (EMFACS-7) was later suggested to determine whether basic emotions had prototypical facial expressions [38]. On the other hand, such archetypal utterances are uncommon in ordinary life. Instead, small changes in one or a few identifiable facial features, such as tightening of the lips in anger or obliquely dropping the lip corners in melancholy, are more typically used to transmit emotion [39].

Other works, such as [40, 41], have provided FER automatically based on FACS. Face and eye detection, which includes facial landmarks, head attitude, and eye gaze, is the first step in automatic recognition. Then, using a classifier, AU estimation is performed.

To the authors' knowledge, [15, 16], and [17] are the only studies that exclusively address audio-visual deepfake detection using emotive cues. To detect falsifications or manipulations in the input video, the approach provided in [15] concurrently utilizes the audio (speech) and video (facial) modalities, as well as perceived emotion components collected from both modalities. They used both modalities to detect similarity (or dissimilarity) between modality signals, and they discovered that perceived emotional information aids in detecting deepfake content. [16, 17] offered a technique for detecting deepfakes based on semantic consistency in emotion, which was based on previous emotion identification work that extracted emotions over time from a subject's speech and faces separately. Synthesized voices or faces are then detected by analyzing these emotional signals.

## 2. Methods

The materials utilized in this study, including how they were created, are described in this section. Considered facial action units for emotion recognition are then specified. Finally, the procedure and design are described, including the models used to analyze the materials, how they are evaluated, and which metrics are employed.

### 2.1. Materials

The Celeb-DF database includes 590 genuine videos and 5639 DeepFake videos in the Celeb-DF, encompassing over two million photograms. With a normal frame rate of 30 frames per second, the average length of all videos is around 13 seconds. The real videos were selected from publicly available YouTube videos showing interviews with 59 celebrities of various genders, ages, and ethnic backgrounds. Furthermore, the real films show a wide range of changes in factors, such as the size of the subjects' faces (in pixels), orientations, lighting conditions, and backgrounds. DeepFake films are made by switching the faces of each of the 59 subjects. The finished videos are in MPEG4.0 format [35].

Celeb-DF's videos were created with a DeepFake synthesis algorithm based on the original DeepFake maker framework [19]. This framework is based on variational autoencoders (VAEs) and generative adversarial networks (GANs). It consists of 6 subnetworks, including two domain image encoders, two domain image generators, and two domain adversarial discriminators. The algorithm learns translation in both directions in one shot, and it was improved in different ways to address the following unique visual artifacts that have been detected in prior datasets:

- Enhancement of the simulated face to 256 × 256 pixels.
- The color disparity between the synthesized donor's face and the original target's face has been significantly reduced.
- Improved face mask creation phase by synthesizing a face with more surrounding context to cover the original facial parts after warping completely. The result is a smoother mask based on landmarks on the eyebrow and interpolated points on the cheeks and between the lower lip and the chin.
- Reduced temporal flickering by integrating temporal correlations among the observed facial landmarks.

### 2.2. Facial Action Units in Emotion

Face feature extraction is a critical step in recognizing accurate facial expressions. The FACS approach introduced by Ekman and Friesen [36] has been the beginning point for encoding facial traits. The facial action coding system (FACS) [36] explains facial expressions through action units (AUs), which are physically tied to the contractions of specific facial muscles and can occur singly or in combination. EMFACS [38], based on FACS, was designed with a subset of AUs associated with emotions in mind, as described in the previous section. It is possible to map AUs onto the fundamental emotion categories using a finite number of criteria, as described in the FACS Investigators' Guide [42]. The action units (AUs) and categorical emotions associated with them are shown in Table 1. Happiness (Ha), Sadness (Sa), Surprise (Su), Fear (Fe), Anger (An), and Disgust (Di) are the categorical emotions. Faces that lacked any emotion-related AUs were classified as Neutral.

### 2.3. Procedure and Design

The convolutional experts network model [43] is used in the OpenFace facial analysis tools [38]. An input image is given, and a region of interest of size *n* × *n* is retrieved from it based on the estimated landmark position. This small region is passed via a contrast normalizing convolutional layer with a kernel shape 500 × 11 × 11 that performs Z-score normalization before the correlation operation, resulting in a 500 × *n* × *n* with *n* = *n*−10. The response maps are then fed into a 200 × 1 × 1 convolutional layer containing ReLU units. The mixture of expert layer employs a convolutional layer of 100 × 1 × 1 sigmoid probability decision kernels to develop an ensemble to capture ROI fluctuations. The output response map is a nonnegative and nonlinear combination of neurons in the mixture of expert layer using a sigmoid activation. The convolutional experts network model was used to extract a set of facial features for each frame of each video in the Celeb-DF database. These features include face location and rotation, gaze direction, the location of face parts in 2D and 3D, the presence or absence of AUs, and the intensity of AUs if they are present.

Two possible AU prediction models exist, depending on dynamism. The static one uses a single image to estimate the existence or intensity of AUs (henceforth, the static model). In contrast, the dynamic model is calibrated to a person by performing person normalization in the video and seeking to adjust for over and under prediction of AUs (subsequently, the dynamic model). By default, static models are used for photos and dynamic models for image sequences and videos. However, some video sequences have limited dynamic range (the same expression is maintained throughout the clip), making postcalibration ineffective and possibly damaging [41]. We applied both prediction models to analyze the input videos and compared the results.

The total number of photograms with faces analyzed was 225,390 for original videos and 2,116,768 for fake videos in the Celeb-DF database. Obtained data was saved to files in CSV format, including original and fake videos in the static and dynamic models, adding up to 23,4 GB of data to be analyzed.

After all videos from the Celeb-DF database were analyzed with both static and dynamic models, we developed a script to select the set of emotionally relevant photograms for each model. This selection was based on the mapping of AUs onto the basic emotion categories described by Ekman using the rules displayed in Table 1. The selected set added up to 7,6 GB of data.

Subsequently, the selected set was analyzed using the following metrics:

- The percentage of fake recordings with emotional photograms per performer and emotion
- For each emotion, the number of emotional photograms that are only in the original, only in the corresponding fake, and those in both
- Sample data and detected overall emotion (amount and percentage) per performer for dynamic and static models, considering emotions just in the original, the fake, and those in common

## 3. Results

Table 2 describes the detected emotional recordings in the fakes in the sample, as detected emotions vary considerably between performers in the database. It shows the percentage of recordings with emotional photograms in the Celeb-DF database for each emotion and performer, both in dynamic and static models. The recordings with emotional photograms are displayed by their percentage normalized to the number of fake recordings per performer. For example, the 14.04% in Sadness for performed with id0 means that the 14.04% of fake recordings of the performer with id0 included at least one sad photogram with the dynamic model. Most relevant values in each column (the highest values) are displayed in bold.

Table 3 shows the results for recognized emotional photograms in the Celeb-DF database. The outcomes for each emotion are shown by the number of emotional photograms in common (EPC), emotional photograms in original alone (EPOA), and emotional photograms in fakes alone (EPFA). Photograms in common are the ones that share a given emotion by the original recording and the corresponding photogram of a fake recording made on the original one. In contrast, original alone photograms are the ones that showed emotion in the original but not in the fake, and fake alone are the ones that showed emotion in the fake but not in the original one. The percentages are also shown by the percentage of common emotional photograms (%C), emotional photograms in original alone (%O), and emotional photograms in fakes alone (%F). To adequately display the data, emotional photograms detected in original recordings were split between the ones in common with the corresponding fakes and those just in the original. Therefore, values in columns of common and original alone for each emotion sum 100%. The percentage of fakes is calculated compared to the sum of common and original alone photograms. As both dynamic and static models were used in the study, both are included. The results shown are global, including all recordings in the database, and they intend to show similarity in emotivity between original and fake recordings. Most relevant values in each column (the highest values) are displayed in bold.

**Figure 1:**
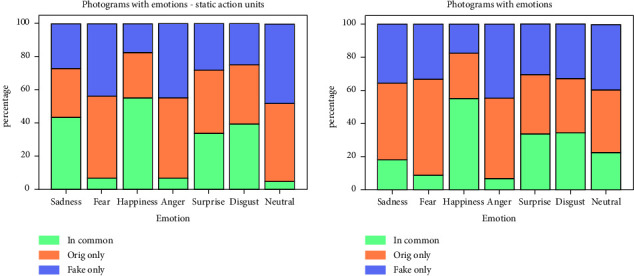
Percentages of emotional photograms in common, original alone, and fakes alone, displayed by emotion (static model on the left, dynamic model on the right).

Figure 1 displays the percentages of common, original alone, and fake alone photograms per emotion, both in dynamic and static models. 100% is depicted as the sum of common, original alone, and fake alone. The aim is to properly portray the similarity between emotional photograms in original and fake recordings for each emotion.

Performers' identifiers range from 0 to 61; however, there were no performers with ids 14, 15, and 18. Hence, there are no rows for them. As for the performer with id 36, he is in the original recordings of the Celeb-DF database; however, no fakes were performed because he was wearing glasses.

Table 4 shows information about the sample per performer. The first four columns display information about performer id (Id), number of original recordings by the performer (OR), number of performers of deepfakes on the original recordings of the current performer (PF), and number of fake recordings made on recordings by the performer (FRP). The remaining columns show the number of photograms with emotions in common with the original recording and the corresponding photogram of a fake recording (CO), the number of photograms that showed emotion in the original recording but not in the corresponding fakes (OR), and the number of emotional photograms that appear in fakes but not in corresponding photograms on original recordings alone (FA). Next to them, their corresponding percentages are displayed. Values in columns of common (%CO) and original alone (%OR) sum 100%, and the percentage of fakes (%FA) is calculated compared to the sum of common and original alone photograms, as in Table 3. Some results over 100% appear in fake percentages, as the number of fake recordings is larger than the original ones, and the percentage of fakes is calculated compared to the sum of common and original alone photograms. As dynamic and static models were used in the study, both outcomes are displayed. Most relevant values in each column (the highest values) are displayed in bold.

**Figure 2:**
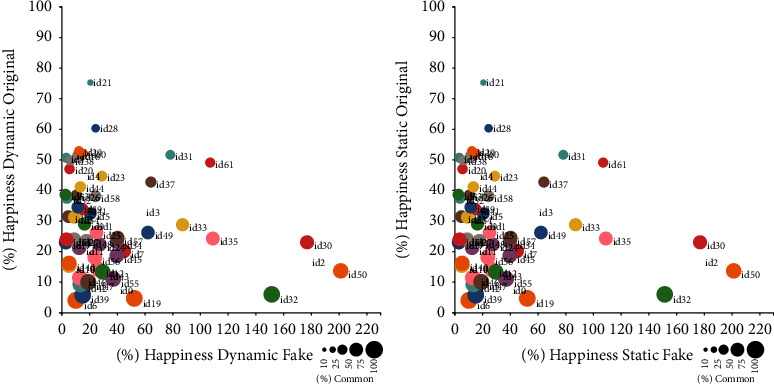
Percentages of emotional photograms in common, original alone, and fakes alone for Happiness emotion, displayed by the performer.

**Figure 3:**
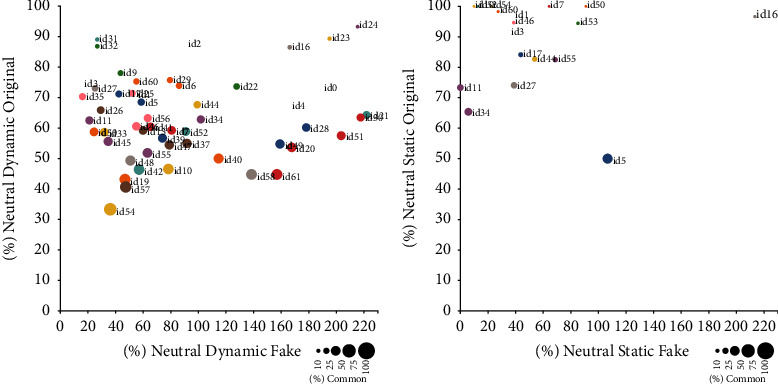
Percentages of emotional photograms in common, original alone, and fakes alone for Neutral emotion, displayed by the performer.

**Figure 4:**
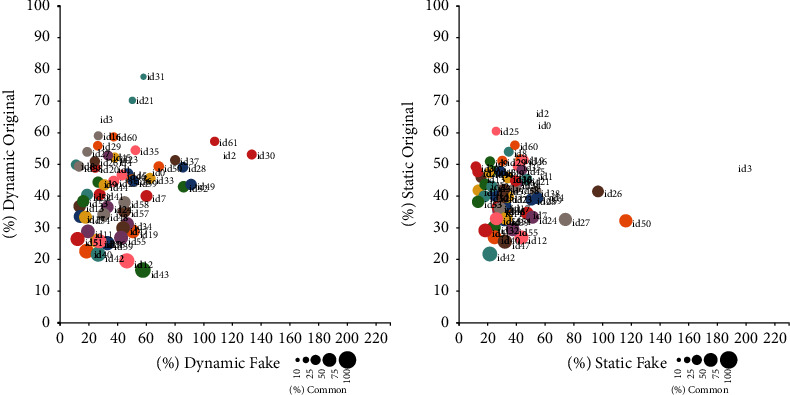
Percentages of emotional photograms in common, original alone, and fakes alone for all emotions together, displayed by the performer.

As the amount of data per emotion was too big to display adequately using tables, we have used figures to represent global data and the most representative two emotions graphically. Figures 2–4 display results for happiness emotion, neutral emotions, and all emotions together. Each figure confronts the results for dynamic and static models. The plots in each figure have an *X*-axis representing the percentage of emotional photograms of the corresponding emotion present just on the original recordings. The *Y*-axis represents the percentage of emotional photograms of the corresponding emotion present on just the corresponding fake recordings. Finally, the diameter of the corresponding circle displays the percentage of common emotional photograms. Therefore, a high percentage in *X* represents a high percentage of emotional photograms in original recordings alone, while high percentage in Y represents a high percentage of emotional photograms in fake recordings alone. The circles with big diameters represent high common percentages between original and fakes. The id of the corresponding performer is placed next to its corresponding circle. At the bottom right part of each image, a scale details the percentages corresponding to the different diameters, which serve as a visual guide. The caption of each figure shows the emotion or emotions it represents.

In some cases, fake recordings showed a specific emotion, and their corresponding originals did not. When that happened for all original recordings of a given performer, as the percentage increase should be performed over 0%, we did not include these recordings in the previous figures. The number of performers in which that happened, for each emotion and model, were as follows:

- Dynamic model: Sadness 4, Fear 13, Happiness 1, Anger 17, Surprise 5, Disgust 1, Neutral 2
- Static model: Sadness 0, Fear 22, Happiness 1, Anger 17, Surprise 2, Disgust 1, Neutral 23

Finally, there were no performers without neutral emotion in both originals and fakes using the dynamic model. However, there were 16 without neutral emotion detections using the static model.

## 4. Discussion

There are no known studies about emotions in deepfake databases. We address this shortcoming by analyzing a database that includes recordings with emotional content and is based on the most used deepfake strategy for deepfakes and face-swapping. Our study focuses on how well emotions are displayed in these databases when faces are swapped between different performers.

The selected deepfakes database, Celeb-DF [35], is a well-known deepfake database whose original videos were chosen from publicly available YouTube videos corresponding to interviews of celebrities. It includes a wide range of facial expressions by the performers. Although it is a fairly new database, it has already been cited many times, over 170 in Scopus and 300 in Google Scholar as of May 2022.

As recordings in the Celeb-DF database were not selected with emotions in mind, we first determined how emotional the fakes used the OpenFace emotion detection tool. Table 2 shows that fake recordings included emotions, regardless of the type of model used to analyze them. The use of two different models was decided because of the difficulty to capture spatial-temporal information of expressions with a slight motion in facial emotion databases [10]. Still, the percentage of fake recordings that included emotions varied notably, considering each specific performer, emotion, and model. Overall, the static model detected considerably more emotional recordings than the dynamic one. Besides, there are significant differences among different emotions. Almost no performer expressed fear in any recording, which is not surprising as it is the most difficult to display, considering it involves more action units (AUs) [36] than other emotions. On the other end, happiness was portrayed by almost all performers.

To properly compare emotions detected in fakes with the originals, we split the percentage of emotional photograms detected in original recordings between those just in the original and those in common with the corresponding fakes. Results in Table 3 show that the metric that indicates the “goodness” of emotions in fakes, the percentage of common emotional photograms between original and fake recordings, is higher than 50% only for Happiness, Disgust, and Sadness just in the case of the static model. The percentage varied widely, between 9.08% and 66.77%, depending on the emotion and the model. The difference in categorical emotion display is consistent with the literature. The difference in emotion recognition is usually shown in the confusion matrices of the results obtained by machine learning systems. Happiness is usually the emotion with better recognition rates, and Fear is the one with the worst in posed expressions [44]. Figure 1 shows that normalizing all detected emotional photograms to 100% per emotion, and these differences are also evident when considering global data. As for the used models, the proportions of emotions are similar in both cases. The only exceptions are for the static model, in which Sadness is noticeably higher and Neutral drops considerably.

In this regard, the results for Neutral photograms stand out. The percentages in common with the corresponding fakes are medium compared to the other analyzed emotions in the dynamic model and the lowest in the static. There were 16 out of 59 performers for which no neutral photograms were detected using the dynamic model, neither for original nor for fake recordings. Besides, data from 23 performers showed no neutral photograms in fakes when none was found in original recordings. Although the number of performers is small compared to the overall number of performers (as it is displayed in Figure 3), the percentage of common neutral photograms is the lowest of all. In contrast, photograms in common are similar or better for all emotions compared to the dynamic model. It indicates that person normalization-based calibration and correction of AUs performed in the static model works better for emotion recognition. However, it does not perform well for neutral emotion.

Most human facial emotion works focus on specific emotion category recognition, and few references are found on the neutral recognition rate. Most times, such information is shown in confusion matrices. When such information is available, results in neutral pictures are generally good, comparable to the best emotions identified in the same works [45].

Regarding facial emotion recognition in video recordings or dynamic pictures, many recent works tackle it using deep learning. Video recordings in selected emotional video recording databases are preprocessed, and prior knowledge about emotional transitions is used when developing deep learning models. Evaluation protocols select specific frames in the video recordings for model training, typically only the video recordings that have one of the labeled emotions and a neutral frame at the beginning [9]. Or neutral expression frames at the beginning and then a different number of frames near the emotional peak of the sequence [10–12]. As it happens with images, in most cases, neutral is not included when detecting emotions in video recordings [13, 46].

Different problems arise when working with facial emotion databases. Usually, all recordings in benchmark databases reflect the same temporal activation patterns. Taking two of the most referenced facial emotion databases as a guide, recordings in the CK + database [37] include transitions from neutral to the peak of emotion, while recordings in the MMI database [47] follow onset-apex-offset temporal segments. While the CK + database is widely used without much trouble or need for preprocessing, the MMI database is more challenging for a series of reasons. First, the subjects perform emotional expressions in a non-uniform way, as different people perform the same expression in different ways. Second, some subjects have a mustache or wear accessories, such as glasses or headcloths. Third, in some recordings, the apex frames are not with high expression intensity [48]. Finally, some recordings are more complicated as they include several different emotional expressions in the same sequence [49].

As a consequence of these challenges, in many cases, specific subsets of recordings are selected with the less troublesome recordings to achieve better recognition rates [12, 49], including discarding the recordings that do not start with entirely neutral photograms [48]. Neutral emotion is not explicitly classified in many works, even though transitions are a crucial factor when identifying emotions, as most databases do not include specific neutral recordings and initial photograms are considered neutral [49]. Besides, the difficulty in capturing spatial-temporal information of expressions with slight motion is a common trait in all facial emotion databases [10].

Neutral is understood as the lack of a specific categorical emotion. In the field of facial AUs, it can be interpreted as the lack of any AUs or the lack of any emotion-related AU, which is the approach we used in this work. Bad results in common neutral percentages indicate that neutrality is not adequately transferred to fakes. It is important to note that emotional video affects recognition systems are mainly based on transitions from neutral to specific emotions. Consequently, the effect of bad neutral photograms in fakes, mainly in initial photograms, may suggest that emotion recognition is prone to work worse than in their corresponding original recordings. Works on how to include emotional cues in deepfake detection are starting to be performed [15–17], which indicates that emotional cues in video recordings can be used effectively for deepfake detection.

Results show that the improved algorithm based on the basic DeepFake maker algorithm [19], used in the Celeb-DF database, does not adequately transfer emotions expressed with facial expressions to deepfakes created from original recordings with emotional photograms. Our study also shows the great extent of the differences between emotions and performers when adequately displaying emotions in performed fakes (see Table 4). We can draw two main conclusions regarding deepfake creation. First, emotions should be considered criteria for selecting original recordings to make fakes. Second, performer selection should also be tuned to performers with similar emotional expressiveness.

Of course, using one deepfake database, even though it is widely used and includes a reasonable amount of original and fake recordings, limits the generalizability of the findings. Therefore, more databases should be considered, their emotional expressivity analyzed, and comparative analysis performed for originals and fakes. Considering static facial expressions has improved the percentages of common detections in original and fake recordings for some emotions, while it has worsened them for others. This difference indicates that mixed approaches should be considered when analyzing emotions in recordings. Moreover, the results suggest that the scope of neutrality, although not discussed enough in the related literature, can be considered a factor affecting achieved results. As for the dynamic and static models used to extract facial expressions, although they are widely used, they may not have adequately identified all AUs. In this regard, the OpenFace implementation was selected because it has been trained and tested against multiple facial expression databases, including databases with emotional recordings [41].

Another aspect to discuss is that presented study has been performed on a database with images with good quality and contrast. In case a database or sample images do not have adequate quality or contrast, the use of contrast enhancement techniques would be necessary for better affective image processing. In these scenarios, the scientific literature offers a wide range of fuzzy image preprocessors that could be used or adapted, such as color extraction methods [50] or fuzzy image preprocessors based on geometric computations in Euclidean spaces [51]. These computations are characterized by a reduced computational load, which is particularly useful for any real-time applications.

## 5. Conclusion

As far as we know, the presented study is the first one that addresses explicitly facial emotion expression in deepfakes. According to the findings, the deepfakes that were produced utilizing a face swap algorithm from the original recordings are not sufficiently capable of recreating emotional expressions. Concretely, a much higher proportion of emotional photograms is found in fake recordings compared to the authentic ones. Additionally, we saw that emotions varied widely from one another. The percentage of times the same feeling was present when a photogram revealed emotion in the false recording and the corresponding original was high. These incidences, however, were insignificant when compared to the percentages of emotional photograms that were only found in genuine or fraudulent recordings. Besides, a high variability has been observed between emotions and performers, while adjustments to face dynamism show a better common emotion recognition between originals and fakes, and worse for neutral. Therefore, performer emotion expressiveness should be considered for better deepfake creation.

The results of this study have ramifications for the development and detection of deep fakes. When constructing fakes from authentic recordings, deepfake algorithms should consider how well they convey emotions. Regarding deepfake identification, our results support the variation in emotional expressions between authentic and fake recordings as a viable data source for identifying fake recordings.

## Figures and Tables

**Table 1:** Action Units related to emotions according to Ekman [36].

Basic emotion	Involved action units
Sadness	AU 1, 4
Fear	AU 1, 2, 4, 5, 7, 20, 26
Happiness	AU 6, 12
Anger	AU 4, 5, 7, 23
Surprise	AU 1, 2, 5, 26
Disgust	AU 9, 15, 16

**Table 2:** Percentages of fake recordings with emotional photograms, displayed by performer and emotion.

ID	Dynamic model							Static model						
	%Sa	%Fe	%Ha	%An	%Su	%Di	%Ne	%Sa	%Fe	%Ha	%An	%Su	%Di	%Ne
id0	14.04	0	10.53	0	38.6	22.81	36.84	53.51	5.26	10.53	0	57.89	42.11	0
id1	19.61	0	49.67	0	9.8	0.65	60.78	51.63	0.65	49.67	0	8.5	52.29	5.88
id2	4.23	0	12.68	7.75	19.72	9.15	21.13	47.18	0.7	12.68	7.75	42.25	0	0
id3	27.46	0	31.69	0	7.75	30.28	54.93	89.44	0	31.69	0	21.83	10.56	2.11
id4	38.89	0	23.02	0	44.44	24.6	19.84	92.06	5.56	23.02	0	39.68	53.17	0
id5	16.67	0	33.33	0	77.78	50	44.44	44.44	0	33.33	0	94.44	55.56	5.56
id6	18.49	0	17.65	0	17.65	23.53	59.66	90.76	0	17.65	0	21.01	62.18	0
id7	17.24	0	41.38	0	3.45	37.93	79.31	55.17	0	41.38	0	48.28	65.52	6.9
id8	0	0	60	0	56	0	0	80	0	60	0	60	24	0
id9	4.21	0	47.37	0	0	21.05	55.79	74.74	0	47.37	0	0	28.42	0
id10	0	0	40.63	0	59.38	56.25	56.25	25	0	40.63	0	68.75	18.75	0
id11	45.45	0	63.64	0	36.36	36.36	45.45	63.64	0	63.64	0	45.45	81.82	0
id12	36.36	0	81.82	0	54.55	9.09	18.18	45.45	0	81.82	0	63.64	63.64	0
id13	13.04	0	39.13	0	17.39	4.35	30.43	30.43	0	39.13	0	82.61	56.52	0
id16	12.32	0	36.45	0	4.93	15.76	32.02	73.4	0	36.45	0	15.76	62.07	3.45
id17	5.47	0	25.78	0	0	9.38	26.56	28.13	0	25.78	0	10.16	44.53	13.28
id19	6.94	0	20.83	0	6.94	5.56	38.89	20.83	0	20.83	0	13.89	63.89	0
id20	19.89	0	30.11	0.54	10.22	0	22.04	61.83	9.14	30.11	0.54	27.42	55.38	0
id21	10	0	13.89	0	0	13.33	38.89	77.22	0	13.89	0	0	45	0
id22	0	0	0	0	8.7	2.17	6.52	34.78	0	0	0	21.74	36.96	0
id23	27.08	0	47.92	14.06	12.5	8.85	9.9	77.08	0	47.92	14.06	10.42	73.96	0
id24	15.19	0	68.35	0	41.77	21.52	20.25	39.24	0	68.35	0	30.38	54.43	0
id25	32.2	0	27.12	0	15.25	20.34	22.03	76.27	0	27.12	0	61.02	20.34	0
id26	14.96	0	30.71	0	5.51	13.39	59.84	59.06	14.96	30.71	0	62.2	14.17	0
id27	1.45	0	20.29	0	18.84	7.25	69.57	26.09	0	20.29	0	13.04	43.48	8.7
id28	14.46	0	8.43	0	28.92	10.84	30.12	54.62	0	8.43	0	38.96	35.34	0
id29	13.25	8.43	38.55	0	61.45	56.63	21.69	42.17	19.28	38.55	0	89.16	65.06	0
id30	23.63	0	56.04	2.75	0.55	4.95	35.16	58.24	7.14	56.04	2.75	23.63	43.96	0
id31	17.77	0	12.69	0	1.52	24.87	19.8	82.23	0	12.69	0	24.37	56.85	0
id32	10.17	0	61.02	0	13.56	52.54	16.95	94.92	0	61.02	0	16.95	91.53	0
id33	29.36	0	68.81	0	15.6	35.78	33.03	90.83	0.92	68.81	0	43.12	77.06	0
id34	21.67	0	68.33	18.33	45	38.33	6.67	91.67	0	68.33	18.33	55	78.33	1.67
id35	0.55	0	29.12	7.69	36.26	9.89	43.96	58.24	0	29.12	7.69	43.41	48.9	2.2
id37	23.9	0	22.64	0	0	5.66	19.5	81.76	0	22.64	0	1.26	61.64	0
id38	10.94	0	39.06	6.25	4.69	7.81	0	53.13	0	39.06	6.25	42.19	31.25	0
id39	13.64	0	18.18	3.03	48.48	10.61	48.48	50	0	18.18	3.03	53.03	56.06	0
id40	1.2	0	57.83	0	18.07	7.23	46.99	27.71	1.2	57.83	0	78.31	55.42	0
id41	7.81	0	67.19	0	21.88	17.19	56.25	34.38	0	67.19	0	42.19	90.63	0
id42	13.89	0	25	0	22.22	8.33	47.22	50	0	25	0	41.67	22.22	0
id43	0	0	60.76	0	20.25	18.99	36.71	8.86	0	60.76	0	50.63	31.65	0
id44	0	0	73.08	0	0	26.92	26.92	61.54	0	73.08	0	15.38	65.38	15.38
id45	26.58	0	29.11	0	8.86	17.72	84.81	31.65	0	29.11	0	37.97	70.89	0
id46	0	0	16.67	1.67	15	11.67	85	13.33	0	16.67	1.67	80	65	10
id47	1.12	0	30.34	0	15.73	5.62	71.91	60.67	0	30.34	0	37.08	58.43	0
id48	19.05	0	55.95	0	33.33	21.43	63.1	40.48	0	55.95	0	36.9	69.05	0
id49	32.47	0	66.23	0	28.57	5.19	54.55	55.84	1.3	66.23	0	80.52	72.73	0
id50	18.31	0	63.38	0	25.35	19.72	76.06	59.15	0	63.38	0	46.48	59.15	7.04
id51	21.88	0	50	0	51.56	43.75	18.75	51.56	0	50	0	70.31	82.81	0
id52	31.65	0	48.1	5.06	35.44	16.46	41.77	77.22	5.06	48.1	5.06	51.9	87.34	0
id53	15.38	0	57.69	0	35.9	26.92	47.44	43.59	0	57.69	0	33.33	75.64	5.13
id54	21.25	0	50	0	33.75	28.75	61.25	50	0	50	0	38.75	62.5	7.5
id55	0	0	84.38	0	40.63	46.88	76.56	46.88	4.69	84.38	0	70.31	68.75	6.25
id56	6.33	0	88.61	0	56.96	35.44	39.24	31.65	0	88.61	0	75.95	94.94	0
id57	0	0	42.86	0	32.14	23.21	62.5	21.43	0	42.86	0	57.14	26.79	0
id58	6	0	56	0	42	26	64	32	0	56	0	70	56	0
id59	34.78	0	30.43	0	69.57	47.83	30.43	56.52	0	30.43	0	69.57	78.26	4.35
id60	30	0	76.67	26.67	53.33	53.33	70	96.67	16.67	76.67	26.67	76.67	93.33	26.67
id61	6.67	0	20	0	60	46.67	80	40	0	20	0	86.67	33.33	0

Happiness (Ha), Sadness (Sa), Surprise (Su), Fear (Fe), Anger (An), and Disgust (Di) are used for dynamic and static models.

**Table 3:** Global data of emotions in original and fake recordings, displayed by emotion.

Emotion	Dynamic model						Static model					
	EPC	EPOA	EPFA	%C	%O	%F	EPC	EPOA	EPFA	%C	%O	%F
Sadness	6047	15099	11827	28.60	71.40	55.93	242246	166103	150972	59.32	40.68	36.97
Fear	46	295	171	13.49	86.51	50.15	349	2513	2232	12.19	87.81	77.99
Happiness	190312	94701	61022	66.77	33.23	21.41	190312	94701	61022	66.77	33.23	21.41
Anger	431	3029	2808	12.46	87.54	81.16	431	3029	2808	12.46	87.54	81.16
Surprise	13378	14196	12078	48.52	51.48	43.80	77445	87270	64647	47.02	52.98	39.25
Disgust	13106	12685	12583	50.82	49.18	48.79	181323	163951	115179	52.52	47.48	33.36
Neutral	36971	61327	64760	37.61	62.39	65.88	197	1973	2031	9.08	90.92	93.59

**Table 4:** Sample data and detected overall emotions.

ID	OR	PF	FRP	Dynamic model						Static model					
				CO	OR	FA	%CO	%OR	%FA	CO	OR	FA	%CO	%OR	%FA
id0	10	13	114	2608	2406	3026	52.01	47.99	60.35	5029	8427	7012	37.37	62.63	52.11
id1	10	17	153	8943	8529	6405	51.18	48.82	36.66	12674	11117	12469	53.27	46.73	52.41
id2	10	17	158	1652	1871	3866	46.89	53.11	109.74	5515	10780	8253	33.84	66.16	50.65
id3	10	17	157	3868	6988	2691	35.63	64.37	24.79	4409	4280	16585	50.74	49.26	190.87
id4	10	15	140	3939	4129	3001	48.82	51.18	37.2	11665	7909	11677	59.59	40.41	59.66
id5	10	3	18	508	452	453	52.92	47.08	47.19	2323	1568	959	59.70	40.30	24.65
id6	10	17	134	3718	3055	3386	54.89	45.11	49.99	15487	8694	7760	64.05	35.95	32.09
id7	10	4	29	1167	779	1170	59.97	40.03	60.12	3419	1811	2479	65.37	34.63	47.40
id8	10	9	33	2990	2980	661	50.08	49.92	11.07	3486	4096	2626	45.98	54.02	34.63
id9	10	17	124	5582	4460	2652	55.59	44.41	26.41	8850	9186	3892	49.07	50.93	21.58
id10	10	4	33	3155	1101	1089	74.13	25.87	25.59	3376	2428	785	58.17	41.83	13.53
id11	11	1	11	877	356	238	71.13	28.87	19.3	1686	926	719	64.55	35.45	27.53
id12	7	2	11	941	229	543	80.43	19.57	46.41	1559	576	928	73.02	26.98	43.47
id13	16	2	23	2169	1263	465	63.2	36.8	13.55	3422	2904	974	54.09	45.91	15.40
id16	14	16	203	5853	8438	3792	40.96	59.04	26.53	23137	19710	14574	54.00	46.00	34.01
id17	10	16	143	8448	4272	1773	66.42	33.58	13.94	10427	6117	5311	63.03	36.97	32.10
id19	10	9	72	2342	951	1667	71.12	28.88	50.62	3234	3449	2858	48.39	51.61	42.77
id20	10	27	212	7194	6925	3443	50.95	49.05	24.39	33724	30693	8398	52.35	47.65	13.04
id21	10	27	205	1486	3504	2508	29.78	70.22	50.26	12585	10429	10840	54.68	45.32	47.10
id22	10	9	52	96	139	615	40.85	59.15	261.7	4371	3393	1415	56.30	43.70	18.23
id23	10	27	218	5353	5838	4246	47.83	52.17	37.94	24505	16198	13878	60.20	39.80	34.10
id24	10	9	79	4402	2536	2280	63.45	36.55	32.86	6526	3265	5013	66.65	33.35	51.20
id25	11	9	59	1584	1279	1067	55.33	44.67	37.27	3596	5512	2352	39.48	60.52	25.82
id26	10	27	170	5075	5278	2501	49.02	50.98	24.16	6988	4953	11561	58.52	41.48	96.82
id27	10	9	77	2147	2517	878	46.03	53.97	18.83	2951	1430	3247	67.36	32.64	74.12
id28	10	27	249	3236	3125	5438	50.87	49.13	85.49	15921	10156	13149	61.05	38.95	50.42
id29	10	13	103	2901	3679	1722	44.09	55.91	26.17	10211	10678	6299	48.88	51.12	30.15
id30	10	21	201	2731	3096	7776	46.87	53.13	133.45	40638	39511	9427	50.70	49.30	11.76
id31	10	21	197	1372	4774	3566	22.32	77.68	58.02	39716	30033	16023	56.94	43.06	22.97
id32	10	13	88	1435	1081	2163	57.03	42.97	85.97	16032	6920	5593	69.85	30.15	24.37
id33	10	13	109	4217	3536	4852	54.39	45.61	62.58	18643	15752	11702	54.20	45.80	34.02
id34	10	13	87	4654	2113	3162	68.77	31.23	46.73	7265	5690	5222	56.08	43.92	40.31
id35	10	21	182	4653	5571	5360	45.51	54.49	52.43	15156	14880	12323	50.46	49.54	41.03
id37	10	21	172	3197	3374	5264	48.65	51.35	80.11	32742	21484	16629	60.38	39.62	30.67
id38	10	13	104	3032	2957	790	50.63	49.37	13.19	8002	5657	7295	58.58	41.42	53.41
id39	10	8	72	3694	1242	1620	74.84	25.16	32.82	10710	5169	5005	67.45	32.55	31.52
id40	10	9	83	7527	2201	1783	77.37	22.63	18.33	12327	4566	4189	72.97	27.03	24.80
id41	10	8	71	3497	2381	1616	59.49	40.51	27.49	9380	5408	4002	63.43	36.57	27.06
id42	5	9	36	3505	968	1184	78.36	21.64	26.47	3994	1109	1095	78.27	21.73	21.46
id43	10	9	79	5443	1088	3761	83.34	16.66	57.59	9751	8985	4371	52.04	47.96	23.33
id44	6	9	46	1861	1435	1013	56.46	43.54	30.73	2820	2540	1139	52.61	47.39	21.25
id45	10	9	87	3411	3814	2408	47.21	52.79	33.33	4296	4011	3640	51.72	48.28	43.82
id46	10	9	76	1945	1702	1573	53.33	46.67	43.13	3233	3416	2993	48.62	51.38	45.01
id47	10	9	89	3884	1660	2426	70.06	29.94	43.76	14475	4996	6221	74.34	25.66	31.95
id48	10	9	84	5035	2619	2322	65.78	34.22	30.34	10434	5901	4678	63.88	36.12	28.64
id49	10	9	84	2406	1865	3894	56.33	43.67	91.17	9071	8312	5011	52.18	47.82	28.83
id50	10	8	71	3302	3216	4478	50.66	49.34	68.7	4821	2292	8265	67.78	32.22	116.20
id51	10	8	67	11224	4040	1850	73.53	26.47	12.12	17190	7082	4431	70.82	29.18	18.26
id52	10	8	79	8931	6069	2812	59.54	40.46	18.75	21332	14171	6285	60.09	39.91	17.70
id53	10	9	86	9810	6115	2557	61.6	38.4	16.06	17295	10690	3707	61.80	38.20	13.25
id54	10	9	80	11734	5870	3106	66.66	33.34	17.64	13703	6736	6694	67.04	32.96	32.75
id55	10	9	64	7576	2783	4395	73.13	26.87	42.43	10837	4537	5743	70.49	29.51	37.36
id56	10	9	79	9131	3168	3366	74.24	25.76	27.37	14968	7330	5804	67.13	32.87	26.03
id57	10	9	63	4874	2652	3366	64.76	35.24	44.72	6896	5005	3735	57.94	42.06	31.38
id58	10	8	55	4436	2726	3208	61.94	38.06	44.79	6122	4539	4179	57.42	42.58	39.20
id59	10	3	23	1036	840	955	55.22	44.78	50.91	3038	1975	2735	60.60	39.40	54.56
id60	10	3	30	1862	2647	1682	41.3	58.7	37.3	3853	4919	3415	43.92	56.08	38.93
id61	10	3	15	360	483	907	42.7	57.3	107.59	1796	1459	1285	55.18	44.82	39.48

Columns display the number of original recordings by the performer (OR), the number of performers of deepfakes on the original recordings of the current performer (PF), and number of fake recordings made on recordings by the performer (FRP). Then, the number of photograms with emotions in common with the original recording and the corresponding photogram of a fake recording (CO), the number of photograms that showed emotion in the original recording but not in the corresponding fakes (OR), and the number of emotional photograms that appear in fakes but not in corresponding photograms on original recordings alone (FA) are displayed followed by their corresponding percentages, for dynamic and static models.

## Data Availability

The tabular data used to support the findings of this study have been deposited in the CORA repository (https://doi.org/10.34810/data262).
